# Magnesium Recovery from Nanofiltration Brine by Membrane Distillation Crystallization

**DOI:** 10.1021/acssusresmgt.5c00219

**Published:** 2025-08-31

**Authors:** Asif Saud, Aamer Ali, Cejna Anna Quist-Jensen

**Affiliations:** † Center for Membrane Technology, Department of Chemistry and Bioscience, 1004 Aalborg University, Fredrik Bajers Vej 7H, 9220 Aalborg East, Denmark; ‡ AAU Energy, Aalborg University, Pontoppidanstræde 111, 9220 Aalborg East, Denmark

**Keywords:** circular economy, critical mineral, co-crystallization, brine management, desalination

## Abstract

Membrane distillation crystallization (MDCr) is gaining recognition as a sustainable and cost-effective method for treating hypersaline brine. The current study explores magnesium sulfate (MgSO_4_) crystallization by using MDCr from synthetic nanofiltration (NF) brine. The study evaluates three feed temperature conditions (41.8 °C, 54.9 °C, and 64.5 °C), along with the corresponding permeate temperatures (19.9 °C, 23.2 °C, and 26.2 °C) and flow rates (1.3 and 0.7 L/min). The tested conditions revealed that temperature impacts the MDCr performance and MgSO_4_ crystallization more effectively than the flow rate. The presence of other ions (Na^+^, K^+^, and Cl^‑^) decreases the solubility of MgSO_4_ (compared with the theoretical solubility at the tested temperature) and increases the tendency of co-crystallization with NaCl, which poses a significant challenge in the final separation stage. The examined process conditions (feed temperature 64.5 ± 0.5 and flow rate 1.3 L/min) successfully delay the crystallization of MgSO_4_, toward a higher water recovery factor (65.98 %), owing to the higher solubility of MgSO_4_ at higher temperatures, which minimizes the extent of co-crystallization. The recovered crystals (a mixture of NaCl and MgSO_4_) are then separated by selectively dissolving NaCl in a saturated solution of MgSO_4_. No compromise with the permeate purity (<5 μm/cm) was observed under all tested conditions.

## Graphical abstract

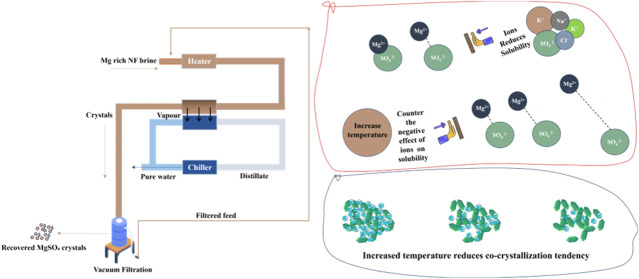

## Introduction

Seawater reverse osmosis (SWRO) accounts for nearly 70% of the global desalination capacity.^1,2^ Out of all valuable elements, recently, magnesium (Mg) has received scientific and political attention all over the world.^3^ Mg is recognized as a critical raw material due to its industrial importance and high supply risk, and the EU’s Critical Raw Materials Act aims to diversify sources.^3^ Various approaches have been explored for Mg extraction from seawater and brine, including CO_2_ mineralization,^4^ electrodialysis,^5^ and reactive crystallization.^6^ Although these methods show promise, many are energy-intensive and costly and lack precise control over crystallization, often relying heavily on chemical additives. Precipitation is a common method for magnesium recovery from brines, using agents like NaOH, NH_4_OH, Na_3_PO_4_, and Ca­(OH)_2_.^7^ However, these agents can cause issues such as sludge production, unwanted calcium salt precipitation, hazardous ammonium ions, and filtration challenges.^8^ Magnesium can be extracted nonchemically through crystallization as magnesium sulfate, a compound widely used in agriculture,^9^ medicine,^10^ and cosmetics.^11^

MgSO_4_ extraction from brine does not require chemicals but rather an evaporative concentration of the solution to achieve reasonable supersaturation for crystallization. The biggest hurdle to achieve this is the presence of co-existing ions that precipitate before and with MgSO_4_ and disrupt the process and purity. To address this issue, the most reasonable and reliable approach is to remove the unwanted ions, and process integration presents the most feasible solution for achieving this goal. In recent years, several new process integrations for mineral recovery have been proposed.^12–14^ The border scheme of these integrations is the pretreatment of the feed by removing unwanted ions and then concentrating the feed solution for the recovery of target minerals. Hence, in this work, we integrated SWRO (seawater reverse osmosis)–NF (nanofiltration)–MDCr (membrane distillation crystallization). To eliminate co-existing ions, the most appropriate and cost-effective method is the inclusion of NF in the integration. Much work has been done to produce or test NF membranes with high divalent ion (Mg^2+^ and Ca^2+^) rejection and monovalent (Li^+^, Na^+^, and K^+^) permeation. MDCr, the technology that can concentrate the feed solution beyond the solubility limit of the dissolved ions, has attracted significant attention due to its energy-efficient operation, environmental sustainability, and dual functionality in recovering both minerals and high-purity water from hypersaline brines. One of the most typical advantages of MDCr lies in its ability to exert precise control over the crystallization process, ensuring high crystal quality and purity.^15^ The process operates on a temperature gradient, which can be effectively driven by low-grade or waste heat sources, further enhancing its energy efficiency.^16^ Regarding the economic feasibility of the technology, some studies suggest that revenue generated from salt and water production could potentially offset the overall costs associated with the operation, highlighting the promising economic perspective of this technology.^17–19^

Hence, recent studies have highlighted the versatility of MDCr in the recovery of valuable minerals, such as lithium,^20^ rubidium,^21^ sodium,^22^ and magnesium,^17^ from complex brine matrices. For example, the first study by Drioli et al.^23^ treated NF brine for crystallizing epsomite. The experimental work focused on recovering CaCO_3_, NaCl, and MgSO_4_·7H_2_O as solid products from nanofiltration retentate. Mariah et al.^24^ studied the MDCr for concentrated epsomite solutions and mixed solutions with sodium chloride, focusing on sodium chloride crystallization. Quist-Jensen et al.^25^ proposed an RO–MD–MDCr system for mineral extraction, using a polyvinylidene fluoride (PVDF) membrane for epsomite crystallization. A study by Abdel et al.^26^ and J. M. Arnal et al.^27^ utilized evaporative crystallization, and the proposed method required less energy but was time-intensive and did not support water recovery. Additionally, previous MDCr studies have primarily focused on either single-salt solutions, such as MgSO_4_ or a mixture of MgSO_4_ and NaCl, which do not represent the complex nature of brine. On the other hand, most studies have investigated the process under constant operating conditions, particularly a fixed feed temperature, and have not thoroughly explored the phenomenon of co-crystallization or the influence of co-existing ions on the crystallization of MgSO_4_ and the membrane distillation crystallization (MDCr) process under varying temperature conditions.^23–25^ These studies primarily aimed to establish a proof-of-concept and state-of-the-art method for recovering MgSO_4_ using MDCr. Consequently, the critical issue of co-crystallization and its cause remains unexplored, motivating the present work to specifically target this knowledge gap. Overall, this study addresses the significant research gaps identified in the prior literature. Hence, we examined the impact of brine temperature (41.8–64.5°C) and flow rate (0.7-1.3 L/min) on the MDCr performance, solubility, crystallization behavior, and co-crystallization mitigation. By combining the effects of temperature and hydrodynamics, this study provides a detailed understanding of the crystallization process in MDCr, offering critical insights for optimizing conditions to achieve MgSO_4_ recovery while enhancing the overall process performance.

## Materials and Methods

### Synthetic Nanofiltration Brine (Feed Solution)

The feed solution was prepared by mimicking the study conducted by Figueira et al.^28^ The study considered a 35000 m^3^/day capacity per day SWRO plant with a recovery of 40% and NF with a water recovery capacity of 65%. The concentrations utilized in this study are presented in Table 1. A concentration factor of 8x was selected based on the initial salt concentrations. Additionally, the detailed information regarding the PHREEQC simulation can be found in our previous work with the same NF feed.^29^

**Table 1:** Concentration of Ions Used in the Current Study

ions	concentration (ppm)
Ca^2+^	21.7
Mg^2+^	54171.3
SO_4_^2–^	123929.2
Cl^‑^	112496.1
Na^+^	49813.5
K^+^	2747.1

### MDCr Setup and Process

The experimental setup utilized a lab-made hollow fiber module consisting of 25 capillary polypropylene (PP) membranes (0.2 μm pore size, 1.8 mm inner diameter, 450 μm thickness, 73 % porosity) from 3M Membrana GmbH. Heating and cooling systems (Grant Instruments Ltd and Julabo 200F, respectively) and a peristaltic pump (1.7 to 2900 mL/min; Masterflex L/S) were used at equal flow rates. Water activity was measured by an Aqualab 4te (AquaLab Decagon Devices, Inc.) Figure 1 illustrates the general scheme of the lab-scale MDCr setup. Five temperature sensors were installed at key locations: the feed inlet, feed outlet, permeate inlet, permeate outlet, and within the feed tank. The permeate weight was recorded during the tests using a digital balance (A and D, FZ-3000i; *d* = 0.01 g). The permeate flux in the MDCr experiment was calculated as follows

1 $$J=\frac{\Delta w}{\Delta t\times A}$$

**Figure 1:**
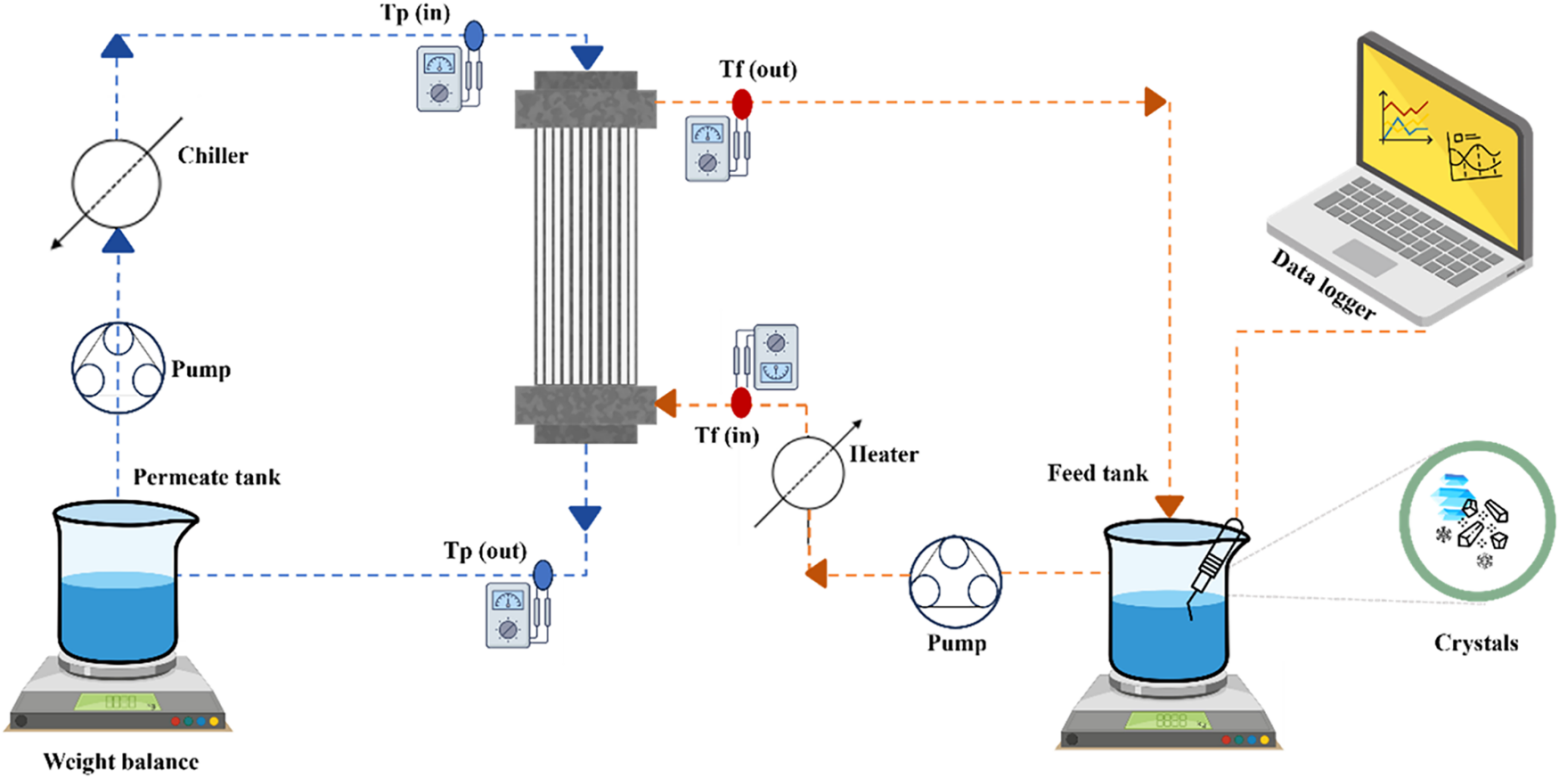
Schematic illustration of the MDCr system used in this study. A direct contact membrane module was used with the feed flowing on the shell side and permeate inside the fibers. Both permeate as well as feed weights were measured by a digital balance. An inline camera was installed in the feed tank to measure turbidity. Two peristaltic pumps were used for circulating the feed as well as permeate. *T*_f_ (in), *T*_f_ (out), *T*_p_ (in), and *T*_p_ (out) represent the feed inlet and outlet temperatures and permeate inlet and outlet temperatures, respectively, which were measured by a digital thermostat.

The parameters Δ*w*, Δ*t*, and *A* represent the collected permeate volumes (ml) since the permeate in MDCr is pure water, the density is assumed to be 1 g/mL for the purpose of converting the collected mass (g) to volume (ml), time interval (h), and membrane surface area (m^2^), respectively. The water recovery factor is defined as

2 $$\mathrm{WRF}\left(\%\right)=\frac{{\mathrm{Vol}}_{\mathrm{permeate}}}{{\mathrm{Vol}}_{\mathrm{initial feed}}}\times 100$$

where Vol_permeate_ is the volume of water recovered as permeate, and Vol_initialfeed_ is the initial volume of the feed solution. In this study, CV% was calculated by using eq 3, where *L*_*x*%_ is the cumulative percent function given by the crystal length at the indicated percentage. CV% is a statistical parameter used to assess the uniformity of the crystal size distribution. When crystal dimensions are evaluated in relation to their corresponding cumulative percentiles (in the current study at *L*_20%_, *L*_50%_, and *L*_80%_), CV% serves as a fine indicator of the size variation within the population, providing critical insight into the consistency of nucleation and growth processes in MDCr.^30^

3 $$\mathrm{CV}\left(\%\right)=\frac{L_{80\%}-L_{20\%}}{L_{50\%}}\times 100$$

The cumulative percent function describes the percentage of crystals at or below a given size, where *L*_20%_, *L*_50%_, and *L*_80%_ correspond to the crystal lengths at the 20th, 50th, and 80th percentiles, respectively. To do this, the *c*-axis (length) of 100 crystals was measured and analyzed by using the PERCENTILE.INC (in Excel) function. For example, *L*_20%_ refers to the crystal length below which 20% of the crystals are smaller and 80% are larger, and follows the same for *L*_50%_ and *L*_80%_. These values were derived directly from crystal size data. A lower CV% indicates precise control, while a higher CV% suggests growth variability.

### Experimental Procedure

The MDCr system was tested at feed temperatures of 42.7 ± 0.6, 54.9 ± 0.6, and 64.5 ± 0.5 °C with corresponding permeate temperatures of 19.9 ± 0.3, 23.2 ± 1.2, and 26.2 ± 3.1 °C, at a constant flow rate of 1.3 L/min to assess the effects of temperature on the performance and MgSO_4_ crystallization. The reported “average feed temperature” refers to the arithmetic mean of the inlet feed temperature (*T*_fin_). Additional experiments at two flow rates (0.7 and 1.3 L/min) and a constant feed temperature (65 °C) were conducted to evaluate the hydrodynamic influence. This approach aims to optimize the operating conditions and evaluate the combined effects of temperature, flow, and brine composition on Mg recovery. All readings were recorded after a 10-minute stabilization period.

### Crystal Analysis

Crystallization was monitored in-line using a ParticleView V19 microscope (METTLER TOLEDO) for real-time crystal growth and turbidity. A Zeiss Axiolab 5 optical microscope with an Axiocam 208 color camera was used for crystal imaging. About 2 mL of the crystal-containing solution was collected, transferred to a preheated test tube, and spread on a glass plate for imaging. Images were analyzed using ImageJ software to measure the crystal size and shape. Evaluating the CSD%, CV%, and mean size is vital for assessing crystal quality and economic value. These parameters were calculated from 100 crystals. In the final step, sodium chloride was removed using saturated MgSO_4_ at room temperature, which dissolved NaCl while retaining magnesium sulfate. The recovered crystals were dissolved in pure water and analyzed for ion composition via ICP-OES (Optima 8000, PerkinElmer)

### Crystal Recovery Procedure

The system was run until MgSO_4_ crystallization occurred, with crystals recovered four times, as indicated by the dotted lines in Figures 2 and 3. These lines mark the WRF% at which recovery and membrane cleaning were performed. The system was halted, and the feed solution was filtered through a 0.45 μm vacuum filter. To prevent further crystallization, the filtrate container was heated on a hot plate. The filtered solution was then reused. Considering the system dead volume, the feed water was carefully removed and collected before filtration. Turbidity signals were used to determine the optimal stopping points for washing and crystal recovery.

**Figure 2:**
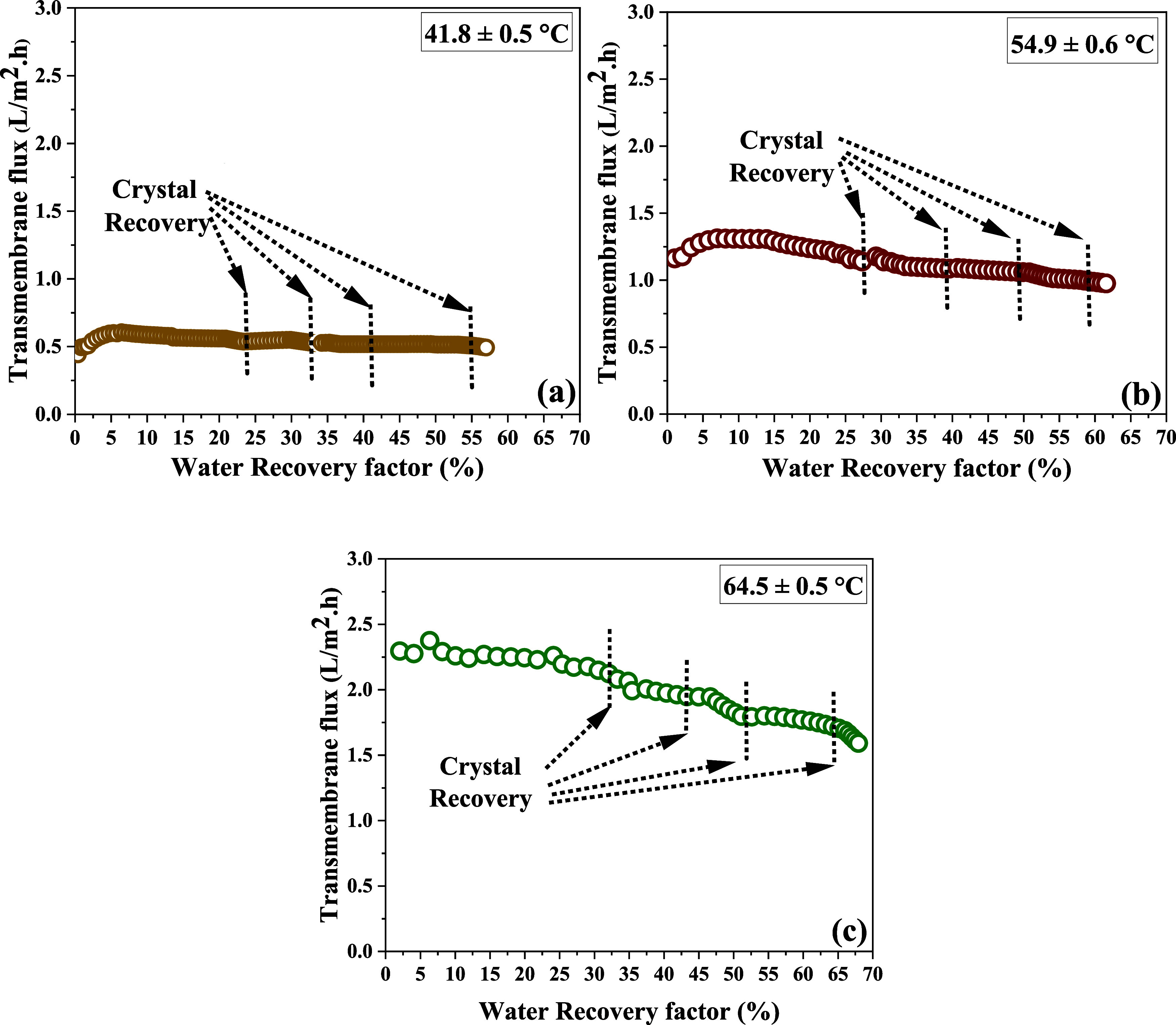
Transmembrane flux at three different feed temperatures: (a) 41.87 ± 0.5 ^o^C, (b) 54.9 ± 0.65 ^o^C, and (c) 64.5 ± 0.53 ^o^C at a constant flow rate of 1.3 L/min. The dotted lines represent the specific WRF% at which the crystals were recovered and the system was washed.

**Figure 3:**
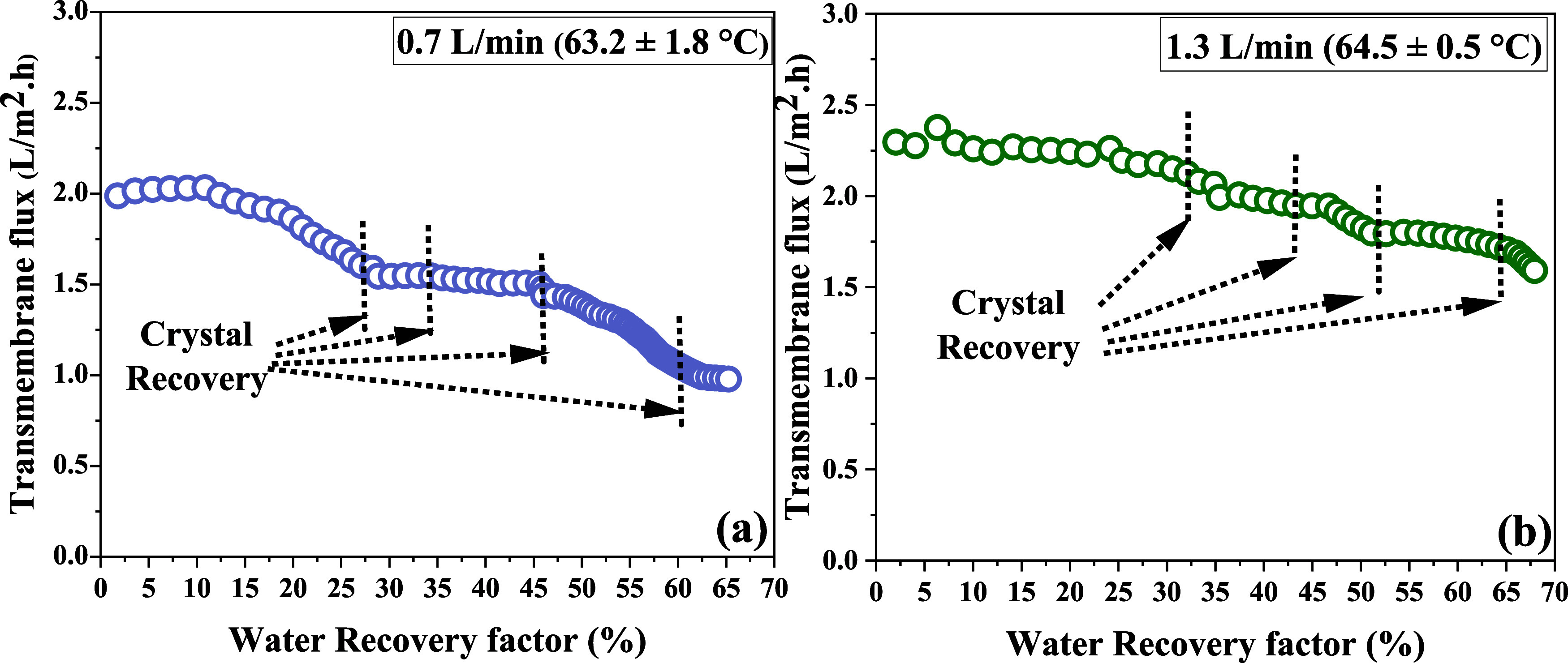
Transmembrane flux at two different flow rate conditions: (a) 0.7 L/min and (b) 1.3 L/min at a constant feed temperature of 65 ^o^C.

### Membrane Washing

After each crystal recovery, the membrane was cleaned with 3 wt % citric acid for at least 2 h and then rinsed with warm distilled water. The cleaning effectiveness was verified by running pure water for 60 min under the same conditions and measuring the transmembrane flux. In all cases, over 98% of the original flux was restored, confirming effective membrane cleaning.

## Results and Discussion

### Effect of Temperature on Membrane Distillation Crystallization

Synthetic nanofiltration brine was tested at three feed temperatures (42.7, 54.9, and 64.5 °C vs 19.9, 23.2, and 26.2 °C) permeate temperatures at a constant flow rate of 1.3 L/min It was observed that at a higher feed temperature (64.5 °C), a higher average transmembrane flux (1.94 ± 0.24 L/m^2^·h) was achieved (Figure 2a). Conversely, at a lower feed temperature (42.7 °C), the average transmembrane flux (0.5 ± 0.03 L/m^2^·h) was reduced (Figure 2c). This trend aligns with the thermodynamic principle that a higher temperature gradient leads to an increased vapor pressure difference, thereby enhancing the driving force for water transport across the membrane.^20,24^ As the feed solution approaches saturation and eventually supersaturation, the transmembrane flux decreases accordingly. As per the observation, the transmembrane flux decreases by 34.03% and 18.09% from higher to lower feed input temperature conditions. It is important to note that these percentages were calculated by considering the highest and lowest flux values.

On the other hand side it was expected, as previous studies suggested that the transmembrane flux should recover to a higher value, surpassing the flux value at which the system was stopped for crystal recovery and washing.^21,22,31^ However, the results presented here show a different pattern. For instance, at a feed temperature of 64.5 ± 0.5°C, the transmembrane flux (Figure 2a) before membrane washing was 1.99 L/m^2^·h at a WRF of 31.41%; after washing, it was 2.00 L/m^2^·h within 10 min, but then began to decline again. A similar trend was observed at other temperatures.

At 54.9 ± 0.6 °C, the flux was 1.13 L/m^2^.h and became 1.17 L/m^2^·h (WRF of 27.94%) and then decreased continuously (Figure 2b). Similarly, at 42.7 ± 0.66°C, the flux was 0.53 L/m^2^·h and became 0.54 L/m^2^·h (WRF of 23.99%) after 10 min and then dropped again (Figure 2c).

The consistent pattern observed, where transmembrane flux did not increase but instead decreased after each stage of membrane washing, provides strong evidence that the decline in flux is primarily driven by a reduction in water activity rather than scaling or salt deposition alone. The reduction in water activity was primarily driven by the increasing Mg^2+^ concentration. Mg^2+^, with its smaller crystal radius and entropy of hydration among all available ions, forms a compact hydration shell, tightly structuring water molecules around its charge.^32^ As water evaporates and NaCl precipitates, Mg^2+^ becomes the dominant ion, governing the bulk solution’s properties, as reported by Guan et al.^33^ This reduction in water activity directly impacts the driving force for permeation, and the calculated water activity is reduced from 0.76 to 0.64, 0.77 to 0.65, and 0.79 to 0.62, corresponding to increasing temperature and respective WRF%. This finding aligns with the observations reported by other authors.^22,34^

It is essential to emphasize that approximately 60% pure water recovery was consistently achieved under all three tested conditions. The permeate conductivity in each case remained below 5 μS/cm, clearly highlighting the advantage of MDCr in facilitating effective crystallization while simultaneously producing high-quality pure water. This result firmly establishes MDCr technology as more reasonable than reactive crystallization methods (e.g., recovery of Mg as Mg­(OH)_2_), where no water recovery is possible^6,35^

### Effect of Hydrodynamics on MDCr Performance

MDCr experiments were conducted at two flow rates (0.7 and 1.3 L/min) to evaluate the effects of hydrodynamics on the MDCr and crystallization of MgSO_4_.

At a flow rate of 1.3 L/min, the average transmembrane flux was 1.94 ± 0.24 L/m^2^·h, with a flux decline of 34% (Figure 3a). When the flow rate was reduced to 0.7 L/min, the average feed temperature dropped slightly to 63.27 ± 1.8 °C, resulting in a lower average transmembrane flux of 1.42 ± 0.32 L/m^2^·h and a higher flux decline of 52.22% (Figure 3b). These results highlight the strong sensitivity of the transmembrane flux and its dependence on the flow rate. A low flow rate not only reduces the average transmembrane flux but also leads to a more pronounced decline, emphasizing the critical role of operating conditions in optimizing MDCr performance. It is important to note that the permeate conductivity under all conditions was less than 5μS/cm. When the flow rate was reduced from 1.3 to 0.7 L/min, the MDCr performance in terms of transmembrane flux (Figure 3a,b) and average feed side temperature was reduced. In the MDCr process, heat transfer occurs in three stages: from the bulk feed to the membrane surface, through the membrane, and from the membrane surface on the permeate side to the bulk of the permeate.^36^ A slight decrease in the average feed temperature (64.5 ± 0.54 °C to 63.7 ± 1.8 °C) was observed at a lower flow rate despite maintaining the same heater set point. This is due to the longer residence time of the feed inside the pipes before reaching the membrane modules, which leads to a greater temperature drop, as heat is transferred to the environment because of conductive losses through the pipes.^37^ At lower flow rates, temperature and concentration polarization are also more significant, further decreasing the driving force for mass transport across the membrane.^38^ These combined effects, originating from a reduced flow rate, lower the transport of water vapor across the membrane.

### Effect of Temperature and Flow Rate on Crystallization

Varying the feed temperature causes a shift in the crystallization points of MgSO_4_, as shown in Figure 4a–d with values in Table 2, which can be attributed to its positive solubility deviation with temperature.^39^ The solubility of MgSO_4_ is highly temperature dependent; for example, it increases from 750g/L at room temperature to +1100g/L at 60 °C. Consequently, MgSO_4_ crystallizes at different supersaturations and WRF% under different temperature conditions (Table 2). It is important to mention that the crystallization of MgSO_4_ was observed at concentrations below its known solubility at all tested temperatures, indicating a significant reduction in the solubility of MgSO_4_ under the experimental conditions. Although the temperature significantly influences the solubility of MgSO_4_, it is not the only factor. The presence of ions (Na^+^, K^+^, and Cl^–^) in the solution significantly reduces the solubility of magnesium sulfate, resulting in earlier crystallization.

**Figure 4:**
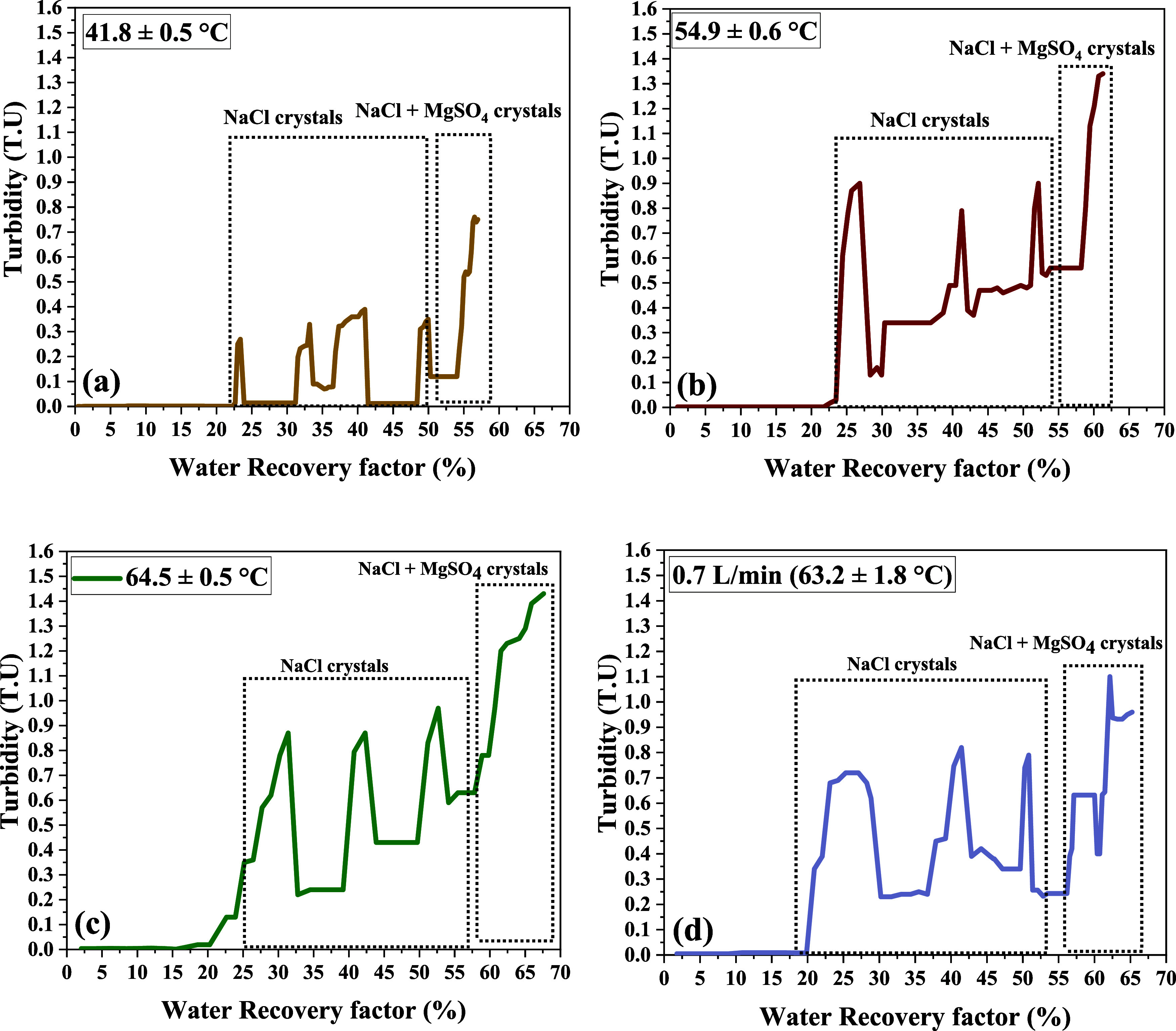
Four turbidity graphs representing the WRF% for crystal recovery at three different temperatures (a–c) and two different flow rates (a, d). The first three peaks in each graph correspond to NaCl crystallization, while the final peak represents NaCl + MgSO_4_ co-crystallization.

**Table 2:** Detailed Description of NaCl and MgSO_4_ Crystallization, Supersaturation, and Point of Crystallization with Reference to Water Recovery Factor^a^,^b^,^c^

temperature (^°^C)	flow rate(L/min)	NaCl crystallization SS (g/L)WRF (%)		MgSO_4_ crystallization SS (g/L)WRF (%)	
64.5 ± 0.5	1.3	236	25.1	905	65.9
63.2 ± 1.8	0.7	236	25.3	876	63.8
54.9 ± 0.6	1.3	235	24.4	780	59.7
41.8 ± 0.5	1.3	230	23.3	708	55.2

a The concentration and water recovery factor correspond to the appearance of the first crystal.

b SS: supersaturation.

c WRF (%): water recovery factor.

The study by Al-Jibbouri,^40^ which was also reported by Drioli^23^ demonstrated that the solubility of MgSO_4_.7H_2_O decreases with increasing individual concentration of NaCl and MgCl_2_ in solution; in particular, the addition of 5 wt % NaCl at 25 °C was reported to reduce the solubility of MgSO_4_ to a level typically observed at approximately 1.4 °C lower temperature in single-salt MgSO_4_ solutions. Hence, the presence of Na^+^ and Cl^–^ ions in NF brine reduces the solubility of MgSO_4_ significantly, promoting co-crystallization with NaCl and resulting in a nonseparable mixture of salts.

However, this condition can be minimized by increasing the feed temperatures. For example, increasing the feed temperature from 41.87 ± 0.5 °C to 64.5 ± 0.5 °C shifts the crystallization point of MgSO_4_, in terms of WRF, from 55.2 to 65.9% (Table 2). This suggests that while the reduction in solubility due to Na^+^, K^+^, and Cl^–^ ions encourages co-crystallization, it can be effectively counteracted by adjusting the feed temperature. By controlling the temperature, the crystallization point of MgSO_4_ can be adjusted as needed.

The NaCl crystals appeared at WRF values of 25.16, 24.41, and 23.37% (Table 2) at corresponding NaCl concentrations of 236.49, 235.84, and 230.32 g/L at all three different feed temperatures (Table 2). It is a well-established fact that temperature has little effect on the solubility of NaCl.^41^ The concentration at different temperatures was reported based on the first crystal appearance under the microscope and confirmed by the turbidity profile (Figure 4a–d). However, the concentration at which NaCl crystals first appeared was much lower than their actual solubility, indicating that the solubility of NaCl decreased significantly in the presence of Mg^2+^, K^+^, Cl^–^, and SO_4_^2–^ ions. This effect can be explained by the Hofmeister series,^42^ which states that both cations and anions play a significant role in the salting-out effect. According to this series, Mg^2+^ and SO_4_^2–^ ions are positioned much earlier than Na^+^ and Cl^–^ ions in terms of their ability to induce a salting-out effect. This means that MgSO_4_ is more effective at reducing the solubility of NaCl than NaCl is at reducing the solubility of MgSO_4_. Therefore, the reduced solubility of NaCl in the presence of co-existing ions, combined with the minimal influence of temperature, allows for greater NaCl recovery earlier in the process. This, in turn, decreases the likelihood of NaCl crystals being included in the final product. The effect of the flow rate on the crystallization of MgSO_4_ was also evaluated. Reducing the flow rate from 1.3 to 0.7 L/min had minimal impact on solubility, as crystallization is primarily driven by supersaturation, which depends on the temperature, which was kept constant in both cases. The slight shift in WRF% observed may be due to increased localized supersaturation at lower flow rates, leading to earlier MgSO_4_ crystallization (Table 2). However, the flow rate had a more noticeable effect on the crystal quality, as discussed in the next section.

### Crystal Characterization

At higher feed temperatures and lower flow rates, narrower CDS% (Figure 5a,b) and low CV% (Figure 5c,d) were observed for MgSO_4_ crystals. At a feed temperature of 64.5 ± 0.54 °C, the mean crystal size was 94.74 ± 32.01 μm. Conversely, as the feed temperature decreased, the CDS% broadened (Figure 5a), and the mean crystal size increased to values of 375.85 ± 49.21 μm at 54.9 ± 0.65 °C and 618.84 ± 62.71 μm at 41.87 ± 0.5 °C. The CV% values followed a similar trend, with higher feed temperatures leading to lower CV% values and vice versa (Figure 5c,d). The larger mean crystal size at lower feed temperatures was because of the low supersaturation state, which promoted slow nucleation and high crystal growth rate.^43^ At lower temperatures, the high CV% and broader CSD% can be attributed to the increased fragility of larger crystals, which are more prone to mechanical breakage under shear forces induced by turbulence and pump roller, leading to significant variability in the particle size distribution.^44^ On the other hand, higher feed temperatures in MDCr lead to a higher rate as well as a state of supersaturation, which promotes a high rate of nucleation as per the classical nucleation theory.^45,46^ Due to the high rate of nucleation, small crystals were produced with a low CV% and narrow CSD%. On the other hand, narrow CSD% and smaller CV% values were observed with decreasing flow rates (Figure 5b,d). The CV% decreased from 24.17 to 20.53 when the flow rate was observed from 1.3 to 0.7 L/min. Hence, it can be stated that a lower flow rate in the MDCr creates a stable and controlled crystallization environment, reducing the variability in crystal size and leading to a lower CV%. At the same time, the flow rate does not have a profound effect on nucleation and crystal growth rate because the crystal kinetics depend on the supersaturation, which is predominantly affected by the temperature.

**Figure 5:**
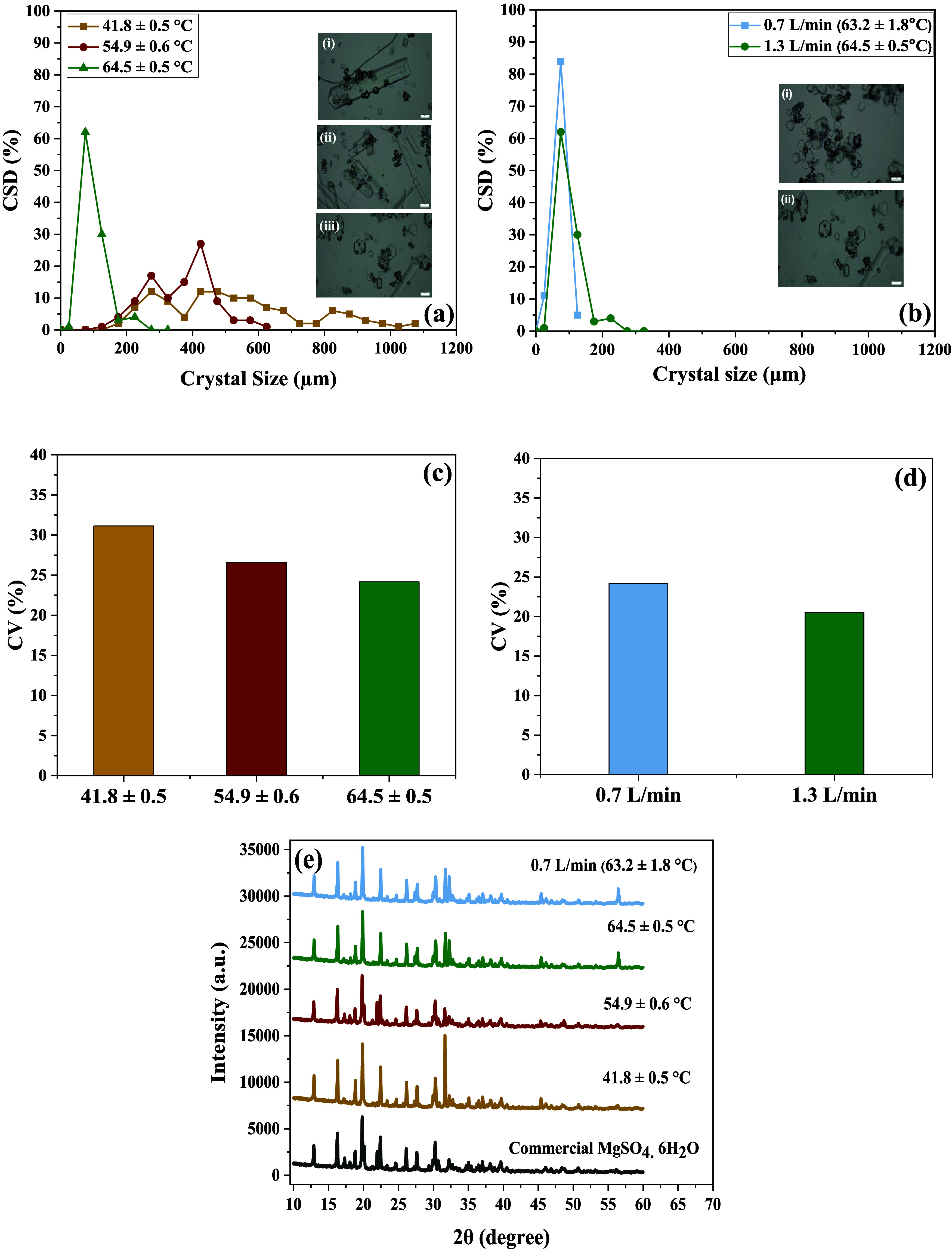
(a) CSD% of recovered MgSO_4_ crystals at different feed temperatures. (b) at different flow rates, (c) CV% at different temperatures, (d) at different flow rates, and (e) XRD of recovered crystals at different temperatures and flow rates.

Producing crystals with low CV% and narrow CSD% is desirable, as it improves downstream processes like separation, storage, and transport.^47,48^ Conventional methods such as MSMPR often yield crystals with CV around 50%,^15^ whereas MDCr consistently achieves lower CV%, as observed in this study. Comparisons further support this: Lu et al. found that vacuum evaporation produced larger crystals with broader size distribution than MDCr,^49^ Jiang et al. reported membrane crystallization yielded larger crystals with lower CV% than conventional cooling,^50^ and Qu et al. observed that vacuum MDC produced more uniform crystals, though with fewer coarse particles, than traditional evaporation methods.^51^

The XRD analysis (Figure 5e) revealed that the major peaks of the recovered MgSO_4_ crystals aligned with hydrated MgSO_4_ (hexahydrate, 6H_2_O),^52^ confirming the absence of impurities within the crystal lattice. Additionally, the peaks for NaCl observed at 2θ = 31.69 ^o^C, 45.39 ^o^C, and 56.50^o^C^53^ appearing in all recovered crystals show the presence of NaCl crystals.

At the end of the process, the crystals were purified, and ICP analysis confirmed the presence of 5.772 g/L Mg^2+^ and 0.0572 g/L Na^+^, resulting in a crystal purity of approximately 99.9%. The minor impurity of about 0.1% Na was attributed to residual NaCl adhering to the crystal surfaces after filtration. However, few relevant studies have discussed MgSO_4_ purity using other crystallization methods. For instance, Kim et al. initially recovered Mg as Mg­(OH)_2_ alongside Ca­(OH)_2_ from RO brine, followed by its conversion to MgSO_4_ using sulfuric acid (H_2_SO_4_). The authors achieved a reported purity of 99.8% by subsequently employing ethanol to reprecipitate MgSO_4_ selectively.^54^ Similar work has been reported by Puthanveettil et al. as well as Na et al., where the authors reported >99% purity of MgSO_4_ by using a multistage precipitation and reprecipitation approach.^55,56^ These approaches utilized a lot of chemicals along with producing a reasonable amount of byproduct (more complex brine) and do not qualify on the merit of the ZLD approach.

## Conclusions

MgSO_4_ crystallization from NF brine via MDCr was evaluated using temperature and flow rate as key parameters. The results show controlled crystallization with a permeate purity of <5 μm/cm. At all tested conditions, it was found that the dominant factor behind flux reduction was water activity, followed by scaling. Out of all the tested process parameters, a feed temperature of 64.5 ± 0.5 °C shows effective performance in terms of MgSO_4_ overall crystallization. Although flow rate variations had a negligible impact on MgSO_4_ crystallization, they influenced the crystal morphology. It was observed that the solubility of MgSO_4_ was significantly reduced in the presence of co-existing ions such as Na^+^, K^+^, and Cl^–^. These ions exerted a salting-out effect, promoting the crystallization of MgSO_4_ at a lower WRF%. Furthermore, the presence of these ions enhanced the co-crystallization of MgSO_4_ with NaCl to a greater extent. While earlier studies primarily focused on the effect of NaCl on MgSO_4_ crystallization, this work also further reports the reverse effect—how MgSO_4_ impacts NaCl crystallization. It was found that increasing the feed temperature can mitigate the negative impact of Na^+^, K^+^, and Cl^–^ ions on MgSO_4_ solubility, which makes temperature a critical parameter for co-crystallization mitigation. At the end, MgSO_4_ was separated from a mixture of MgSO_4_ and NaCl by selectively dissolving NaCl in a saturated solution of MgSO_4_. Lastly, unlike prior works that overlooked the aspect of pure water production, this study successfully demonstrated it as part of the MDCr process.
